# Non-Invasive Diagnosis and Management of Pancreatic *Tetragomphius* sp. Infection in Asian Badgers (*Meles leucurus*) Using Computed Tomography

**DOI:** 10.3390/ani16040577

**Published:** 2026-02-12

**Authors:** Kangyeon Yu, Seri Hong, Sohwon Bae, Woojin Shin, Minjae Jo, Daji Noh, Son-Il Pak, Soo-Young Choi, Sangjin Ahn

**Affiliations:** 1 College of Veterinary Medicine and Institute of Veterinary Science, Kangwon National University, Chuncheon 24341, Republic of Korea; rkddus2401@kangwon.ac.kr (K.Y.);; 2 Gangwon Wildlife Medical Rescue Center, Chuncheon 24341, Republic of Korea

**Keywords:** Asian badger, *Meles leucurus*, pancreatic nodule, *Tetragomphius* sp., computed tomography

## Abstract

Pancreatic lesions in wildlife are rarely diagnosed antemortem and are often misinterpreted as neoplastic due to limited diagnostic information. This report describes two clinically linked cases of pancreatic infection caused by *Tetragomphius* spp. in rescued Asian badgers (*Meles leucurus*) from Gangwon State, Republic of Korea, between 2020 and 2025, and the diagnostic advantages of advanced imaging techniques. The first case involved a badger rescued from a trap with severe traumatic injuries that was subsequently euthanized. Postmortem examination revealed nematodes identified as *Tetragomphius* sp. within the pancreatic duct, alongside tumor-like nodular lesions in the pancreatic tail. These findings were critical for the second case, where a rescued badger with no overt clinical signs underwent computed tomography (CT) that revealed a pancreatic tail nodule and splenomegaly. Drawing on findings from the initial necropsy case, a parasitic etiology was tentatively diagnosed in a second live badger presenting with a similar pancreatic nodule. Follow-up CT indicated a significant reduction (approximately 75%) in lesion size, supporting a diagnosis of parasitic infection. To our knowledge, this represents the first report of a CT-based therapeutic diagnosis of pancreatic *Tetragomphius* infection in a live Asian badger. These cases demonstrate that pancreatic nodules in Asian badgers may result from parasitic infection rather than neoplasia and emphasize the integration of pathological precedent, advanced imaging, and therapeutic response in wildlife medicine.

## Simple Summary

Asian badgers can sometimes develop rare parasitic infections in the pancreas caused by roundworms. These infections may appear similar to tumors, making diagnosis difficult. This report describes two related cases involving rescued Asian badgers in Korea between 2020 and 2025. In the first case, a badger had died, and an examination after death showed clear evidence of worm infection in the pancreas. In the second case, a live badger was found to have a similar lump during a computed tomography (CT) scan. Because the earlier case had already shown a parasitic cause, veterinarians suspected the same problem and started deworming without needing a risky biopsy. Follow-up CT scans showed that the lump had shrunk a lot after treatment, supporting a diagnosis of parasitic infection. These cases show that what seems to be a pancreatic tumor in Asian badgers may actually be a treatable parasitic infection. Using CT imaging, knowledge from earlier cases, and response to medication can help veterinarians diagnose these difficult conditions safely and with less stress for wildlife.

## 1. Introduction

The Asian badger (*Meles leucurus*), a member of the Mustelidae family, is widely distributed across Asia, including the Korean Peninsula [1]. Although these animals are generally elusive, they frequently face anthropogenic threats such as illegal trapping, which poses serious risks to their health and survival [2]. While ecological and behavioral studies on badgers are well-documented, veterinary investigations have primarily focused on infectious diseases, especially bacterial and parasitic infections [3,4].

Among parasitic threats, there are hookworms—hematophagous nematodes that typically reside in the gastrointestinal tract of mammals and can lead to significant morbidity [5]. Despite their recognized pathogenic potential, the diversity, tissue tropism, and clinical implications of hookworm infections in wildlife remain poorly understood [6,7]. Within the Mustelidae family, several hookworm species belonging to the genera *Uncinaria* and *Tetragomphius*, as well as an unidentified *Ancylostoma* species, have been reported [3,8,9].

Notably, *Tetragomphius* spp. represent a distinctive group of hookworms that parasitize the pancreatic duct system rather than the intestine. To date, these parasites have been reported exclusively in Japan and Korea, where *Tetragomphius melis* and *Tetragomphius procyonis* have been identified in badgers [3,6,10,11]. Infections with these parasites are characteristically associated with tumor-like masses in the pancreatic tail, creating a diagnostic challenge and raising the risk of misdiagnosis as pancreatic neoplasia.

This report discusses two clinically linked cases of Asian badgers rescued from illegal traps in Gangwon State, Republic of Korea. The reference case involved a necropsy-confirmed *Tetragomphius* sp. infection of the pancreas, providing essential pathological insight. The index case describes the antemortem detection and therapeutic diagnosis of a pancreatic nodular lesion using computed tomography (CT). Together, these cases highlight the diagnostic value of integrating imaging findings with pathological precedent in wildlife clinical management.

## 2. Case Presentation

### 2.1. Reference Case (Necropsy-Confirmed Pancreatic Tetragomphius Infection)

**Figure 1:**
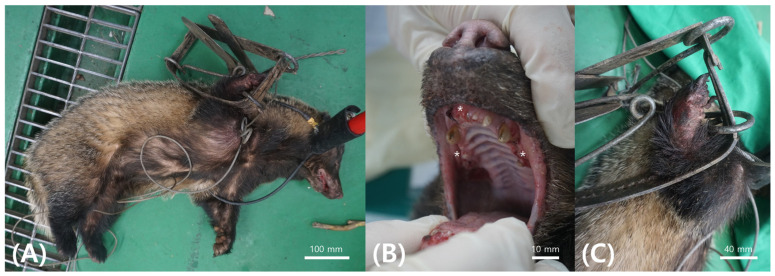
(**A**) Rescue status of an Asian badger, (**B**) severe teeth loss (*), and (**C**) right ulna and radius open fracture due to trapping.

A 7-kg male Asian badger was rescued on 6 April 2020 in Chuncheon, Gangwon State, after being caught in a spring trap and was transferred to the Gangwon Wildlife Medical Rescue Center (WMRC) for clinical evaluation and treatment. Upon rescue, the animal displayed lethargy, dehydration, and severe traumatic injuries, including an open fracture of the right radius and ulna, along with extensive soft tissue damage. Physical examination revealed oral hemorrhage and extensive dental loss, likely sustained during the animal’s efforts to escape the trap (Figure 1).

Hematological and biochemical profiles were compared with reference values for *Meles meles*, as species-specific baseline values for *Meles leucurus* are currently unavailable [12]. When compared with these reference intervals, relative decreases were observed in lymphocyte count, mean corpuscular volume, platelet count, albumin, and alkaline phosphatase, whereas relative increases were noted in white blood cell count, neutrophil and monocyte counts, creatinine, phosphorus, globulin, and blood urea nitrogen (Table A1).

**Figure 2:**
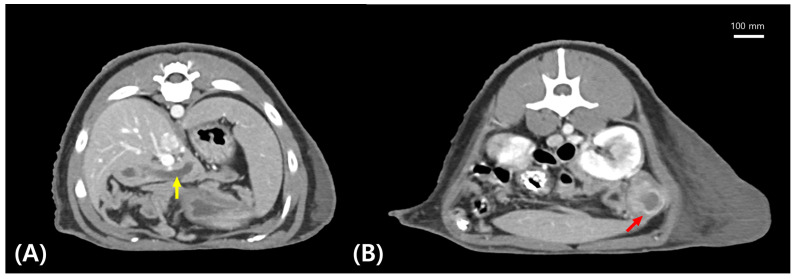
Axial contrast-enhanced computed tomography images showing (**A**) marked dilation of the pancreatic duct (yellow arrow), and (**B**) a well-defined nodular lesion in the left pancreatic tail (red arrow).

**Figure A1:**
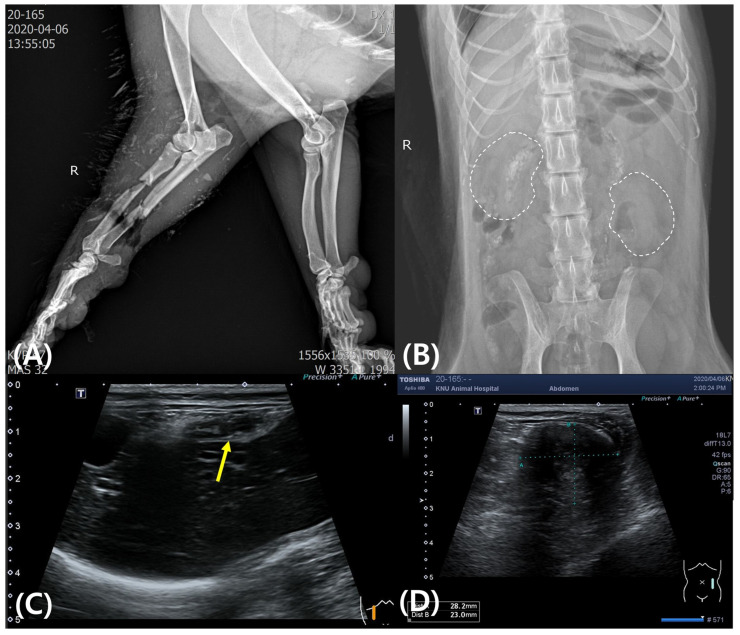
Radiographic findings (**A**) open fracture of right ulna and radius, (**B**) abnormal kidney shadow (dotted line). Ultrasonographic findings (**C**) pancreatic duct expansion (yellow arrow), and (**D**) a mass proximal to the kidney.

Radiography, ultrasonography, and CT were performed. Detailed descriptions of radiographic and ultrasonographic findings are provided in the Table A1 and Figure A1. Radiographic examination revealed fractures of the right radius and ulna, as well as an irregular renal shadow (Figure A1A,B). Ultrasonography indicated pancreatic duct dilation and a mass-like structure in the perirenal region (Figure A1C,D). CT imaging revealed marked dilation of the pancreatic duct and a well-defined 2.6-cm nodular lesion located in the left pancreatic tail (Figure 2).

**CT Protocol:** CT examination was conducted using a Somatom Emotion 6 system (Siemens, Munich, GER). Scan parameters were set at 120 kVp and 150 mAs with a slice thickness of 1 mm. Contrast-enhanced CT was performed using iohexol (Omnipaque™, GE HealthCare, Oslo, Norway) 600 mgI/kg IV, with arterial (5 s), portal venous (35 s), and delayed (90 s) phase acquisitions. Images were reviewed using the image viewing software (ViewRex3, Techheim, Seoul, Republic of Korea), and lesion evaluation and measurements were performed using soft tissue window settings. All procedures involving general anesthesia were performed by licensed veterinarians with appropriate monitoring and supportive care to minimize stress and ensure animal welfare.

**Figure 3:**
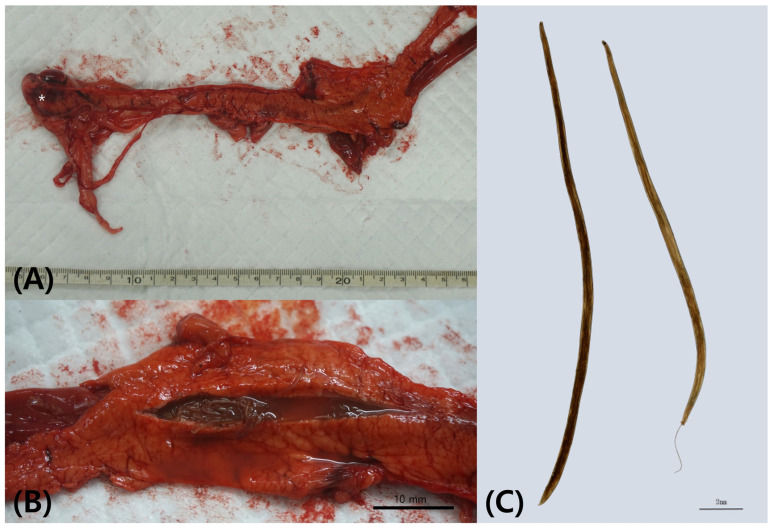
Postmortem examination of the badger revealed (**A**) a tumor-like mass in the pancreatic tail (*), (**B**) parasites in the pancreatic duct, and (**C**) *Tetragomphius* sp. female (left) and male (right).

Due to the severity of injuries, suspected osteomyelitis, and poor prognosis for limb function, euthanasia was performed within one day in accordance with the Badger Rehabilitation Protocol [13]. Postmortem examination revealed pancreatic duct dilation and multiple nematodes within the duct, along with firm, tumor-like nodules in the pancreatic tail (Figure 3A,B).

Morphological examination of the recovered nematodes was performed under stereoscopic microscope (SZN45T-MST2, Sunny, Busan, Republic of Korea). The parasites were examined as whole mounts following fixation without staining or heat killing and were observed in mounting medium under cover slips. Images were captured using a digital CMOS camera (HK6E3 6.3M CMOS Camera, Koptic, Yongin, Republic of Korea) and morphometric measurements were obtained using imaging software (HKBasic Imaging Software v.4.12.28926, Koptic, Yongin, Republic of Korea). Both male and female specimens were identified based on body size and sexual dimorphism. These morphological features, together with their characteristic localization within the pancreatic duct system, were consistent with previously described *Tetragomphius* spp. reported in badgers from Korea and Japan [3,6,10] (Figure 3C).

### 2.2. Index Case (CT-Based Therapeutic Diagnosis of Pancreatic Nodule)

In May 2025, a second Asian badger was rescued from a hiking trail in Yeongwol-gun, Gangwon State, through the same regional wildlife rescue network (WMRC). Although hindlimb lameness was reported at rescue, no overt gait abnormalities were observed upon admission. The animal was alert, with normal appetite and activity. Hematological and biochemical analysis revealed leukocytosis, anemia, thrombocytopenia, hypoproteinemia, hypoglobulinemia, mild hyperbilirubinemia, and hypoglycemia (Table A1).

**Figure 4:**
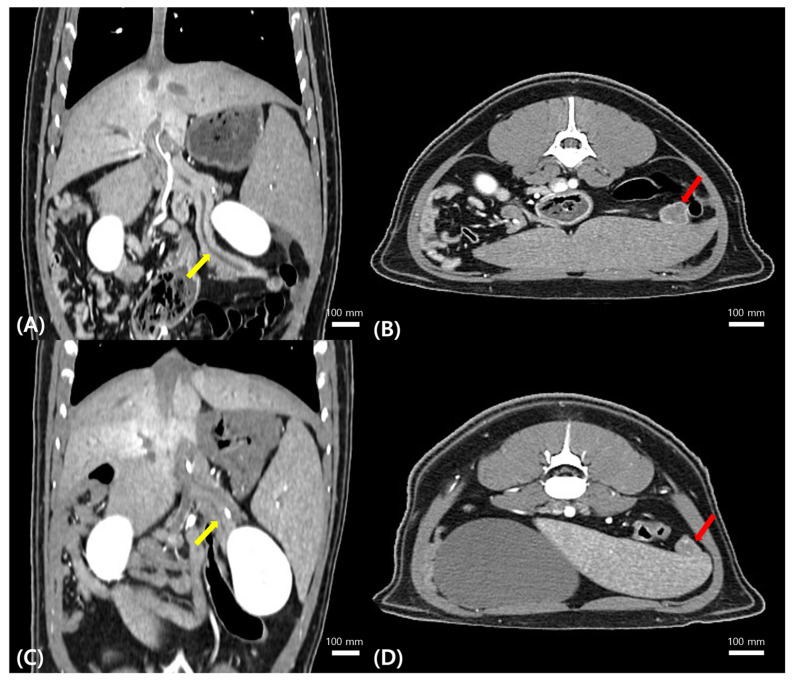
Computed tomographic (CT) findings of the index case. Pre-treatment (**A**) dorsal and (**B**) axial CT images demonstrating dilation of the pancreatic duct (yellow arrow) and a well-defined nodular lesion located in the pancreatic tail (red arrow). Follow-up (**C**) dorsal and (**D**) axial CT images obtained after anthelmintic treatment showing improvement of pancreatic ductal dilation (yellow arrow) and a marked reduction in the size of the nodular lesion in the pancreatic tail (red arrow).

**Figure A2:**
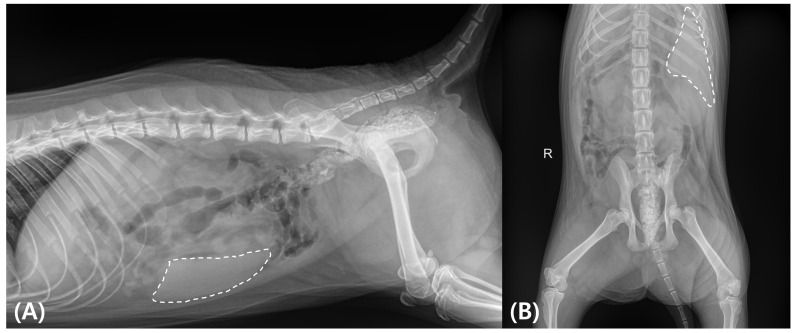
Radiographic findings (**A**) left lateral view, (**B**) ventrodorsal view. There is diffuse enlargement of the spleen, evidenced by increased soft tissue opacity occupying the left cranial to mid-abdominal region (dotted line).

Abdominal radiography suggested splenomegaly (Figure A2). CT examination, performed using the same protocol as in the reference case, revealed splenomegaly and a nodular lesion in the pancreatic tail with imaging characteristics similar to those observed in the reference case (Figure 4A,B).

Based on the prior necropsy-confirmed evidence of pancreatic *Tetragomphius* infection, a parasitic etiology was strongly suspected. Considering the anatomical location of the lesion, the risks associated with invasive pancreatic biopsy in a wildlife patient, and the availability of pathological precedent, a therapeutic diagnostic approach was selected. A presumptive diagnosis of pancreatic *Tetragomphius* infection was made, and fenbendazole was administered empirically (7.5 mg/kg, PO, q24h for four days).

Follow-up CT imaging demonstrated a marked reduction (approximately 75%) in the size of the pancreatic nodule, along with improvement of pancreatic duct dilation (Figure 4C,D). The anthelmintic treatment was discontinued, and the badger remained clinically stable throughout rehabilitation before being successfully released back into the wild.

## 3. Discussion

The pancreatic parasites identified in the reference case were unrelated to the trap injury and were presumed to represent a chronic infection acquired prior to rescue. The morphological features and anatomical localization were consistent with *Tetragomphius* spp. previously reported in Korea [3]. Among the pancreatic-associated *Tetragomphius* species, *T. melis* was first described in Japanese badgers in 1974, while *T. procyonis* was reported in Korean badgers in 2009 [3,10].

Although the pathogenicity and lifecycle of *Tetragomphius* spp. remain poorly understood, the formation of tumor-like nodules in the pancreatic tail appears to be a hallmark feature [6,7]. The reference case provided definitive pathological confirmation, directly informing the diagnostic reasoning for the index case. Species-level identification was not performed in the index case; however, the anatomical localization, imaging characteristics, and therapeutic response strongly supported a diagnosis of *Tetragomphius* spp. infection.

Fecal examination was not performed in either case. Although it is reasonable to assume that eggs or larvae of *Tetragomphius* spp. may be shed in feces, the life cycle, shedding dynamics, and diagnostic utility of fecal examination for this genus remain poorly understood [3,6,7]. No standardized fecal diagnostic criteria currently exist for pancreatic *Tetragomphius* infections, limiting its utility as a confirmatory tool in clinical practice. Therefore, the absence of detectable stages in feces cannot be reliably interpreted as treatment success, and imaging-based monitoring was considered a more objective and clinically applicable approach in this case.

Fenbendazole was selected as the empirical treatment in the index case based on the suspected nematode etiology. *Tetragomphius* spp. are nematodes belonging to the Ancylostomatoidea, for which benzimidazole-class anthelmintics are widely regarded as first-line agents [3,14]. The administered dosage (7.5 mg/kg, PO, q24h for four days) was extrapolated from published anthelmintic protocols used in wild carnivores and mustelids, as species-specific dosing guidelines for Asian badgers are not currently available [15,16]. This dosage falls within the recommended therapeutic range for fenbendazole in wildlife and has been applied to both intestinal and extraintestinal nematode infections [17,18]. Anthelmintics targeting trematodes, such as praziquantel, were not selected because the parasite identified in the reference case was morphologically consistent with a nematode [3]. Although the optimal therapeutic protocol for pancreatic *Tetragomphius* infection has not been established, the marked reduction in lesion size following treatment supports the clinical effectiveness of this empirical approach, which remains presumptive.

The successful resolution of the pancreatic lesion following anthelmintic therapy supports the use of CT as a reliable, non-invasive tool for monitoring treatment response in wildlife species for which invasive biopsy carries substantial anesthetic or surgical risk. In such contexts, therapeutic response itself can serve as valuable diagnostic evidence, helping to prevent misdiagnosis of parasite-associated lesions as neoplasia and to avoid unnecessary invasive interventions [19]. The volume of the pancreatic lesion observed on CT likely reflected not only the physical presence of nematodes within the pancreatic duct but also secondary pathological changes, including ductal obstruction, accumulation of pancreatic secretions, and reactive inflammatory responses such as edema and cellular infiltration [20,21]. Following anthelmintic treatment, parasite death and subsequent relief of ductal obstruction may have facilitated resolution of these secondary changes, resulting in a marked reduction in lesion size [17]. This pattern of regression is more consistent with a parasite-associated inflammatory process than with autonomous neoplastic growth, further supporting the diagnostic value of treatment response in this case [22]. Fenbendazole is known to induce a gradual loss of nematode viability rather than rapid parasite lysis, which may reduce the risk of acute tissue intoxication [14,18]. In addition, the absence of clinical or imaging evidence of pancreatitis following treatment suggests that parasite death and clearance occurred without inducing significant local or systemic toxicity.

A limitation of this clinical evaluation is the reliance on reference intervals from *Meles meles* due to the lack of established baseline values for *Meles leucurus*. While these species are phylogenetically close, further studies are needed to define species-specific parameters for Asian badgers to improve diagnostic accuracy. Histopathological evaluation of the pancreatic nodules was not performed in the reference case, which precludes definitive differentiation between inflammatory and neoplastic processes and is acknowledged as a limitation of this study. Accordingly, alternative explanations, including the possibility of concurrent neoplasia or helminth-induced tumorigenesis, were considered, as such associations have been reported in other systems, including *Opisthorchis felis* infections of the biliary tract [23]. However, several findings support a parasite-associated inflammatory etiology rather than true neoplasia. Most notably, the marked regression of the pancreatic lesion in the index case following anthelmintic therapy, together with concurrent improvement in pancreatic ductal dilation, is more consistent with resolution of parasite-associated obstruction and secondary inflammatory changes than with autonomous neoplastic growth [17,22]. Although an unexpected antitumor drug effect cannot be completely excluded in the absence of histopathology, none of the administered agents are recognized antineoplastic therapies, and the observed imaging response more strongly favors relief of ductal obstruction and inflammation as the primary mechanism of lesion regression.

The abnormal renal imaging findings and multisystemic lesions observed in the reference case were likely attributable to a systemic inflammatory response secondary to severe trap-induced trauma rather than parasitic infection [24]. Spring traps are widely recognized as indiscriminate killing devices that cause severe injury or death in non-target wildlife species [2]. Despite their impact, scientific investigations into the physiological and pathological consequences of trap injuries in Korean wildlife remain limited [25,26].

## 4. Conclusions

This report describes two clinically linked cases of pancreatic *Tetragomphius* spp. infection in Asian badgers, highlighting the diagnostic challenges associated with pancreatic nodular lesions in wildlife. Tumor-like pancreatic masses caused by parasitic infection may closely mimic neoplastic disease, particularly when antemortem diagnostic information is limited. In the present cases, necropsy-confirmed pathological findings from one individual provided essential diagnostic context for the antemortem evaluation of a second case. The use of advanced imaging, particularly computed tomography, combined with a therapeutic diagnostic approach allowed accurate diagnosis while avoiding invasive procedures. These findings emphasize that parasitic infection should be considered in the differential diagnosis of pancreatic nodules in Asian badgers and demonstrate the clinical value of integrating imaging, pathological precedent, and treatment response in wildlife medicine.

## Data Availability

The original contributions presented in this study are included in the article. Further inquiries can be directed to the corresponding author.

## Appendix A

**Table A1:** Hematological and blood chemistry profiles in the Asian badger.

Analyte	Case 1 (Ref.)	Case 2 (Index)	Reference Interval [12]
White blood cell counts (10^9^/L)	9.43	10.4	7.86 ± 4.55
Neutrophils (10^9^/L)	8.23	6.08	6.55 ± 3.96
Lymphocytes (10^9^/L)	0.09	3.57	1.13 ± 0.95
Monocytes (10^12^/L)	1.11	0.76	0.14 ± 0.21
Red blood cell counts (10^9^/L)	10.55	8.02	7.89 ± 1.37
Packed cell volume (%)	41.15	26.43	36 ± 6
Hemoglobin (g/dL)	16.4	10.8	12.67 ± 2.04
Mean corpuscular volume (fL)	39	33	45.95 ± 4.44
Mean corpuscular hemoglobin (pg)	15.6	13.4	16.17 ± 1.72
Mean corpuscular hemoglobin concentration (g/dL)	39.9	40.8	35.19 ± 3.34
Blood urea nitrogen (µmol/L)	7.14	5	12.22 ± 4.77
Creatinine (µmol/L)	97.26	61.89	69.15 ± 31.03
Calcium (mmol/L)	2.1	2.27	2.17 ± 0.22
Total protein (g/L)	79	67	76.42 ± 10.21
Albumin (g/L)	12	38	30.69 ± 6.12
Globulin (g/L)	67	29	45.73 ± 10.84
Alanine aminotransferase (iu/L)	126	41	166.9 ± 127.2
Total bilirubin (µmol/L)	11.97	20.52	12.56 ± 10.11

**Note:** Reference intervals (RIs) for the Asian badger (*Meles leucurus*) have not been established. Therefore, reference values for the Eurasian badger (*Meles meles*) were adopted from Winnacker et al. (2008) [12] for clinical comparison.
